# Osteopontin’s relationship with malnutrition and oxidative stress in adolescents. A pilot study

**DOI:** 10.1371/journal.pone.0249057

**Published:** 2021-03-25

**Authors:** Octavio Gamaliel Aztatzi-Aguilar, Martha Patricia Sierra-Vargas, Manolo Ortega-Romero, Azucena Eunice Jiménez-Corona

**Affiliations:** 1 Departamento de Investigación en Toxicología y Medicina Ambiental, Instituto Nacional de Enfermedades Respiratorias Ismael Cosío Villegas, CDMX, México; 2 Cátedras CONACyT, CDMX, México; 3 Facultad Mexicana de Medicina, Universidad La Salle, CDMX, México; 4 Centro de Investigación y Estudios Avanzados del Instituto Politécnico Nacional, CDMX, México; 5 Escuela Superior de Huejutla, Universidad Autónoma del Estado de Hidalgo, Huejutla, Hidalgo, México; INSERM, Université de Bordeaux, FRANCE

## Abstract

Osteopontin (OPN) is a protein involved in inflammatory illnesses such as fibrosis and cancer; its overexpression in cardiovascular diseases promotes the biomineralization of blood vessels and other soft tissues. Moreover, there is an active component of oxidative stress related with those diseases. The present study relates serum OPN levels with nutritional condition and oxidative stress in a group of adolescents. Anthropometric measurements were performed, and fasting blood samples were analyzed to determine OPN concentrations, blood chemistry parameters (glucose, triglycerides, total cholesterol, urea, uric acid, and creatinine) and oxidative stress biomarkers (Paraoxonase-1, Glutathione S-Transferase, Catalase, NAD(P)H Quinone Oxidoreductase, free carbonyl groups and malondialdehyde). Adolescents were categorized according to body mass index (BMI) and metabolic syndrome (MetS) criteria. We found increased OPN serum concentrations in overweight and obese adolescents, as well as in adolescents with MetS. Rises in OPN correlated with arm circumference and biomarkers of lipid peroxidation; with regard to serum glucose there was a trend to positive correlation. Our results suggest that serum OPN is associated to nutritional status and could be considered as an early biomarker of low-grade inflammation and probably the early biomineralization of soft tissues in adolescence.

## 1. Introduction

Obesity and overweight have become a public health problem worldwide with an increasing prevalence in childhood, ranging from birth to adolescence. The World Health Organization (WHO) estimates that 340 million children and adolescents suffer from overweight or obesity across the globe. In Mexico, the probabilistic national survey ENSANUT MC 2016 estimated a prevalence of obesity during the adolescence was 39.2% in female adolescents and 33.5% in male adolescents [1].

Nowadays, it is known that obesity contributes to insulin resistance via a chronic low-grade inflammation originated from visceral adipose tissue, which increases the risk to develop Type 2 Diabetes Mellitus (T2DM). The detection of early serum circulating molecules could be a good strategy for early diagnosis and, eventually, prevent or delay its complications.

Osteopontin (OPN) is a highly phosphorylated glycophosphoprotein, rich in aspartic acid which confers its acidic characteristics. This ubiquitous protein was first cloned from rat osteosarcoma cells [2, 3] and has recently been related to atherosclerotic vasculopathy in several animal models of diabetes mellitus and chronic kidney disease [4, 5]. Because of the induction of the biomineralization of soft tissues such as the vascular wall, high serum levels of OPN have also been related to coronary artery disease (CAD) in case control studies [6]. Furthermore, increased levels of this protein are associated with poor metabolic control measured by glycated hemoglobin (HbA1c) and subclinical atherosclerosis in pediatric population with type 1 diabetes mellitus [7].

It is known that oxidative stress (OxS) is involved in the pathophysiology of several diseases including respiratory, cardiovascular, obesity, metabolic syndrome, and diabetes mellitus [8–12]. The assessment of OxS and its correlation with several clinical measures could be useful to identify the initial period of a disease and its early complications. Common OxS biomarkers used to evaluate the degree of molecular damage are carbonylation and lipid peroxidation [13, 14]. Antioxidant response is mediated by the induction of non-enzymatic and enzymatic molecules which counteract the effects of OxS. Enzymes such as Catalase (CAT; EC 1.11.1.6), that catalyzes hydrogen peroxide (H_2_O_2_) into water and oxygen, it is present in those sites where H_2_O_2_ is produced in high concentrations; Glutathione-S-Transferase (GST; EC 2.5.1.18) represents a major group of detoxification enzymes, they are implicated in the metabolism of reactive electrophiles and in the reduction of hydroperoxides during lipid peroxidation process and some GST isoforms decrease malondialdehyde and 4-hydroxynonenal (MDA, 4-HNE) [15, 16]. Additionally, the pleiotropic functions of some molecules like circulating high density lipoprotein (HDL) which has intrinsic antioxidant activity due to the paroxonase-1 enzyme (PON-1), have been associated with the development of coronary heart disease and atherogenesis when their concentrations are low [17].

Therefore, the goal of this study, was to determine OPN serum levels in clinically healthy adolescents and whether OPN levels are related with nutritional status and anthropometric measurements, as a reflect of adipose tissue activity and the OxS condition.

## 2. Materials and methods

### 2.1. Subjects selection and study design

Students (N = 35) from 14 to 17 years old were invited and recruited from the General High School of the Universidad Autónoma del Estado de Hidalgo (UAEH), Huejutla de Reyes, Hidalgo State, Mexico. Subsequently, data were collected from 35 confirmed participants; parents and/or tutors signed an informed consent before enrolling in the study. Subjects older than 18 years, with a history of autoimmune diseases (e.g. Type 1 Diabetes), and with immunosuppressive medical treatment were excluded. The study was conducted in accordance with the Declaration of Helsinki and approved by the Committee of Ethics and Research of the Universidad Autónoma del Estado de Hidalgo, with approval number Cinv/ICSa/#060/2018.

### 2.2. Anthropometric data collection

Body weight and height were measured nearest 0.1 kg, and 0.1 m, respectively, with a balance beam-physician scale weight/height rod (Detecto 439). Body Mass Index (BMI) was calculated as weight in kilograms divided by the square of height in meters. Arm and waist circumference were determinated according to WHO [18]. Blood pressure was measured after 5 min rest in the semi-sitting position with a sphygmomanometer. Blood pressure was determined at least three times at the left upper arm, and the mean was used for the analyses. Participants were classified according to body mass index (BMI), as lean (5 < 95th percentil), overweight (85 < 95th percentil) and obese (> 95th percentil).

### 2.3. Blood biochemical analysis

Blood samples were collected after an overnight fast to avoid potential confounding influences due to hormonal rhythmicity. Biochemical evaluation included glucose, urea, creatinine, uric acid, cholesterol and triglyceride levels. Serum glucose was analyzed by an automated analyzer (Roche/Hitachi, Switzerland). Urea, creatinine, uric acid, total cholesterol and triglyceride concentrations were determined by enzymatic spectrophotometric methods (Roche, Basel, Switzerland). Cholesterol/Triglyceride ratio was calculated for predicting the presence of small, dense LDL [12, 19] and Triglycerides/glucose index was calculated to estimate insulin resistance [20, 21].

### 2.4. Assessment serum osteopontin levels

Serum Osteopontin (OPN) concentrations were measured by an enzyme-linked immunosorbent assay (ELISA) using the Human Osteopontin ELISA Kit (Product Number: RAB0436 from SIGMA) and read in a microplate reader (H reader 1, HLAB). OPN concentrations were expressed as ng/mg of total protein.

### 2.5. Free carbonyl groups concentrations

Free carbonyl groups were measured using 50 μl aliquots of serum and 0.5 ml of 10 mM 2,4-dinitrophenylhydrazine (DNPH) in 2.5 M HCl. The absorbance was measured at 370 nm. Carbonyl concentrations were expressed in nmol/mg of protein as described previously [22].

### 2.6. Malondialdehyde concentrations

An aliquot of 100 μl of serum was added to 650 μl of a solution of 1-methyl-2 phenylindole in a mixture of acetonitrile/methanol (3:1). The final concentration of the reagent was 10 mM. The reaction was then started by adding 150 μl of 37% hydrochloric acid. The mixture was incubated 45°C for 40 min and the mixture reaction was measured at 586 nm absorbance [23]. Malondialdehyde (MDA) concentrations were expressed in μM/ mg of total protein.

### 2.7. Paraoxonase-1 activity

Serum Paraoxonase-1 [PON1; EC 3.1.8.1] activity towards paraoxon (O,O-diethyl-O-p-nitrophenyl phosphate) was determined by measuring the initial rate of substrate hydrolysis to p-nitrophenol, whose absorbance was monitored at 405 nm 25 C° in the assay mixture [24]. A PON1 activity of 1 U/mg protein was defined as 1 μmol p-nitrophenol formed/minute adjusted by total protein.

### 2.8. Glutation S-Transferase activity

Serum Glutathione S-Transferase [GST; E.C. 2.5.2.18] activity measures the conjugation of 1-chloro-2,4-dinitro benzene (CDNB) with reduced glutathione that produces a dinitrophenyl thioether which can be detected by spectrophotometer at 340 nm. One unit of GST activity is defined as the amount of enzyme producing 1 mmol of CDNB-GSH conjugate/min under the conditions of the assay according to the method described by Habig et al., enzymatic activity was adjusted by total protein [25].

### 2.9. NAD(P)H:Quinone Oxidoreductase 1 activity

The NAD(P)H:Quinone Oxidoreductase 1 [NQO1 E.C. 1.6.99.2] activity, was performed according with Lind and Höjeberg [26]. The assay was carried out using NADPH (4 mM) as electron donor and 2-methyl-l,4-naphthoquinone (menadione; 0.1 mM) as the electron acceptor. The buffer assay was 50 mM Tris-HCl, pH 7.5 with Triton-100X 0.08%. In order to maintain the redox state of menadiol, cytochrome-c (38.5 μM) was incorporated. Selective assay was performed adding dicoumarol (100 μM, dissolved in 50 mM Tris-HCl, pH 7.5 without Triton-100X). The reaction was started by the addition of the enzyme and followed at 550 nm. Enzyme activity was calculated through the difference between the absorbance of the sample with and without the inhibitor. Slope of each sample was adjusted by the total protein concentration.

### 2.10. Catalase (CAT) activity

Serum Catalase [CAT; E.C. 1.11.1.6] activity was measured according to the method described by Abei [27]. Reaction was performed in 50 mmol/L phosphate buffer pH (7.0). A decrease in the absorbance was recorded at 240 nm. One unit of activity corresponds to the loss of 1 μmol of H_2_O_2_ per minute. CAT activity was calculated by using molar extinction coefficient (ε = 40 mol-1 cm-1) and serum protein concentration was determined by Bradford protein assay. Enzymes activity was expressed as nmol/min adjusted by total protein. All measurements were done at Lambda 25 UV-Vis spectrophotometer Beckman Coulter (DU 800 series).

### 2.11. Statistical analysis

Sample size was estimated for overweight-obese population. According with data of 2016 in Mexico four out of every 10 children between 12 to 19 years old are overweight or obese. Moreover, the prevalence of those conditions is 36.7 (IC 95%: 32.2–41.5) and 35.0 (IC 95%: 29.3–41.1) from urban and rural areas respectively taking into account a population of 18,492, 890 adolescents for that year. Sample size of population with overweight and obesity was nine. The follow equation was used, where N is the population size; e the margin error; z is the confidence level (z = 1.95) [28 we insert this reference to support the sample size calculation].

$$Sample\;size=\frac{\frac{z^{2}*p(1-p)}{e^{2}}}{1+(\frac{z^{2}*p(1-p)}{e^{2}N})}$$

Descriptive analysis were presented as median and 25th-75th percentile range for continuous variables, while category variables were expressed as frequencies and percentages. U Mann-Whitney test was used to compare differences between gender, Kruskal Wallis test followed by Dunn’s multiple comparison post hoc test were used to compare differences among medical body fat conditions. For height, weight, BMI, Systolic Blood Pressure (SBP) and Diastolic Blood Pressure (DBP), Z-score was calculated.

Metabolic syndrome (MetS) was defined as a group of risk factors that predispose to type 2 diabetes, stroke and cardiovascular disease. The MetS categorization was designated with the presence of abnormal values of at least two risk factors (triglycerides, fasting glucose, blood pressure, and abdominal circumference) [29]. Arm circumference was categorized in tertiles to perform statistical comparisons. Spearman correlation coefficient was employed to the relation between OPN and clinical and laboratory variables. Statistical analysis was performed STATA Version 12.0 statistic software package (StataCorp LP). A p value < 0.05 was considered significant in all analyses.

## 3. Results

### 3.1. Population studied

#### 3.1.1. Demographic characteristics

Demographic characteristics of population are showed in Table 1. The median (25th, 75th percentile) age of adolescents was 16 (15–17) years; 57% were female. Abdominal and arm circumference were 83 (74–93.5) cm and 28 (25–30) cm, respectively. Participants were subdivided in normal weight (34.29%), overweight (28.57%) and obese (37.14%) based on the BMI. Arm circumference was subcategorized in three quantiles. Participants were also grouped based on percentile as normotension (48.57%), pre-hypertension (34.29%) and hypertension (17.14%); 51.43% of the participants fulfill the criteria of MetS.

**Table 1:** Descriptive and general characteristics of the population studied.

	Population (n = 35)
	median (25th, 75th percentile)
Age (years)	16 (15–17)
Height (z-score)	-0.55 (-1.53–0.14)
Weight (z-score)	0.9 (0.24–1.68)
BMI (z-score)	1.41 (0.69–1.97)
Abdominal circumference (cm)	83 (74–93.5)
Arm circumference (cm)	28 (25–30)
Waist-size index	0.53 (0.44–0.58)
Systolic Blood Pressure (z-score)	0.66 (-0.47–1.52)
Dyatolic Blood Pressure (z-score)	0.55 (0.39–1.27)
**Gender**	**% (n)**
Male	43 (15)
Female	57 (20)
**Body mass index**	**% (n)**
Normal weight	34.29 (12)
Overweight	28.57 (10)
Obese	37.14 (13)
**Arm circumference**	**% (n)**
1st tertil	37.14 (13)
2nd tertil	42.86 (15)
3rd tertil	20.0 (7)
**Blood pressure (percentile)**	**% (n)**
Normal	48.57 (17)
Pre-Hypertension	34.29 (12)
Hypertension	17.14 (6)
**Metabolic Syndrome (MetS)**	**% (n)**
Without MetS	48.57 (17)
With MetS	51.43 (18)

Values represent median and 25^th^ and 75^th^ percentiles for the first part of the table and percentages and its population´s number for each variable in the second part.

### 3.2. Blood analysis and biomarkers

The biochemical analyses and biomarkers were compared by sex. Statistical differences between sex were not found Table 2.

**Table 2:** Biochemical markers evaluated in adolescents.

	Overall	Male (n = 15)	Female (n = 20)	p-value
	median (IQR)	median (IQR)	median (IQR)	
Glucose (mg/dl)	88 (83–92.5)	85.5 (82.5–91)	89.5 (83–94)	0.81
Urea (mg/dl)	21 (18–28.5)	26.5 (21.5–31)	20.5 (17.5–21.5)	0.10
Creatinine (mg/dl)	0.82 (0.78–0.91)	0.87(0.78–0.94)	0.81 (0.78–0.86)	0.30
Cholesterol (mg/dl)	147 (130–162)	145 (129–170)	149 (130–161)	0.29
Triglycerides (mg/dl)	109.5 (81–167)	105 (81–167)	116 (74–170)	0.30
Chol/TGs ratio	1.19 (0.95–1.6)	1.26 (1.0–1.6)	1.08 (0.87–1.6)	0.34
TGs/Glucose index	8.47 (8.2–8.9)	8.5 (8.23–8.8)	8.4 (8.17–8.9)	0.88
Uric acid (mg/dl)	5.02 (4.6–5.94)	5.26 (4.77–6.39)	4.65 (3.98–5.46)	0.14
OPN (ng/mg of protein)	32.7 (6.7–68.13)	70.05 (23.16–97.42)	30.33(3.94–53.3)	0.107
PON-1 (nmol/mg of protein)	1.22 (0.48–1.83)	1.28 (0.25–1.75)	1.13 (0.63–1.94)	0.44
MDA (μM/mg of protein)	0.018 (0.013–0.21)	0.018 (0.012–0.023)	0.017 (0.014–0.019)	0.50
Carbonyls (nmol osazones/mg protein)	1.18 (0.89–1.36)	1.2 (0.88–1.36)	1.15 (0.98–1.39)	0.86
Catalase (U/mg of protein)	8.9 (3–14.6)	11.3 (4.3–19.5)	7.6 (2.9–13.2)	0.24
GST (nmol/mg of protein)	0.8 (0.7–1)	0.8 (0.7–1)	0.8 (0.7–1)	0.88
NQO1 (U/mg of protein)	49 (38.2–56)	52.6 (41.6–59.7)	46.16 (28.5–53.7)	0.24

Abbreviations: OPN, osteopontin; PON-1, paraoxonase-1; MDA, malondialdehyde; GST, Glutathione S-transferase; NQO1, NAD(P)H:Quinone Oxidoreductase 1; Chol, Cholesterol; TGs, Triglycerides. Mann-Whitney U test; data are shown as the median followed by the interquartile range in parenthesis.

Population characteristics, biochemical analyses and biomarkers were compared based on the body mass index cutoff Table 3. We observed statistical differences in abdominal and arm circumference. Abdominal circumference showed differences between normal-weight and obese, but not with the overweight group. Arm circumference was statistically different among the three groups. With reference to SBP, the obese group displayed the highest SBP among the three groups. On the other hand, obese group showed the lowest concentration of creatinine.

**Table 3:** Clinical and biochemical parameters analyzed based on body mass index in adolescents.

	Overall (35)	Normal weight (12)	Overweight (10)	Obesity (13)	p-value
	median (IQR)	median (IQR)	median (IQR)	median (IQR)	
Age (years)	16 (15–17)	16.5 (16–17)	16 (15–17)	15 (15–16)	0.111
Abdominal circumference (cm)*	83 (74–93.5)	74(66.5–77.25)^a^	85 (73–91.5)^ab^	95(89.5–97.5)^b^	0.0001
Arm circumference (cm) *	28 (25–30)	24 (22–25)^a^	28 (26–30.3)^b^	30 (29–32)^c^	0.0001
SBP (mmHg, z-score) *	0.66 (-0.45–1.54)	-0.33 (-0.89–0.89) ^a^	0.33 (-0.54–1.625) ^ab^	0.92 (0.53–1.73) ^b^	0.028
DBP (mmHg, z-score)	0.55 (0.39–1.27)	0.84 (0.43–1.23)	0.45 (0.32–1.13)	0.55 (0.43–1.33)	0.86
Glucose (mg/dl)	88 (83–92.5)	89 (83–92)	87 (83–93)	85 (83.5–93)	0.75
Urea (mg/dl)	21 (18–28.5)	26.5 (22–32)	20.5 (17–25)	20.5 (17–21)	0.126
Creatinine (mg/dl) *	0.82 (0.78–0.91)	0.92 (0.83–1) ^a^	0.84 (0.81–0.9) ^ab^	0.76 (0.68–0.795) ^b^	0.0081
Cholesterol (mg/dl)	147 (130–162)	154 (135–161)	133 (129–149)	161 (133–170)	0.578
Triglycerides (mg/dl)	109.5 (81–167)	88 (70–167)	102 (74–136)	115 (101–173)	0.867
Chol/TGs ratio	1.19 (0.95–1.6)	0.99 (0.95–1.59)	1.25 (0.98–1.63)	1.15 (0.92–1.59)	0.892
TGs/Glucose index	8.47 (8.21–8.99)	8.45 (8.19–8.99)	8.42 (8.05–8.77)	8.61 (8.4–9.02)	0.445
Uric acid (mg/dl)	5.02 (4.6–5.94)	4.765 (4.65–5.2)	5.3 (4.6–5.79)	5.32 (4.29–6.27)	0.71
PON-1 (nmol/mg of protein)	1.22 (0.48–1.83)	0.98 (0.45–1.86)	1.31 (0.88–1.83)	1.11 (0.29–1.80)	0.60
MDA (μM/ mg of protein)	0.018 (0.013–0.021)	0.017 (0.012–0.023)	0.018 (0.016–0.026)	0.017 (0.011–0.019)	0.56
Carbonyls (nmol/mg of protein)	1.18 (0.89–1.36)	1.19 (0.85–1.44)	1.15 (1–1.43)	1.09 (0.87–1.29)	0.54
Catalase (U/mg of protein)	8.9 (3–14.6)	7.35 (2.55–11.85)	11.35 (3.6–17.8)	10.9 (4.1–16.5)	0.255
GST (nmol/mg of protein)	0.8 (0.7–1)	0.85 (0.75–1)	0.8 (0.7–1)	0.75 (0.65–0.95)	0.61
NQO1 (U/mg of protein)	49 (38.2–56)	50.25 (40.2–59.7)	43.3 (38.2–51.5)	50.9 (28.4–62)	0.99
Gender	n (%) ^b^	n (%) ^b^	n (%) ^b^	n (%) ^b^	
Male	15 (43)	6 (50)	2 (20)	7 (53.8)	
Female	20 (57)	6 (50)	8 (80)	6 (46.2)	

Abbreviations: PON-1, paraoxonase-1; MDA, malondialdehyde; GST, Glutathione S-transferase; NQO1, NAD(P)H:Quinone Oxidoreductase 1; Chol, Cholesterol; TGs, Triglycerides. Kruskal-Wallis test followed by Dunn’s multiple comparisons post hoc test (p<0.05). Different letters indicate statistical differences among groups. Data are shown as the median followed by the interquartile range in parenthesis.

Even though normal-weight group displayed a high level of variability in OPN serum concentrations, the overweight and obese groups showed a statistical difference compared with the former group Table 4.

**Table 4:** Serum osteopontin in adolescents from Huejutla de Reyes, Hidalgo State, Mexico.

Parameter	Normal weight (12)	Overweight (10)		Obese (13)	p-value
**BMI** ^†^	1.99 ^a^	61.63 ^b^		29.48^c^	0.01
	(1.36–66.6)	(31.8–98.5)		(18.5–62.43)	
**Arm circumference (cm)** ^†^	**First tertile (13)**	**Second tertile (15)**		**Third tertile (6)**	
	4.66 ^a^	34.58^a^		92.4^b^	0.018
	(0.47–88.4)	(2.2–98.9)		(23.2–150.7)	
**Metabolic Syndrome** ^‡^	**Without MetS (16)**		**MetS (16)**		
	25.21		44.74*		0.014
	(1.77–116.9)		(24.8–91.06)		

^†^Kruskal-Wallis test; Different letters indicate statistical differences among groups, p<0.05.

^‡^Mann-Whitney U test

* Indicates statistical differences; p<0.05. Data are shown as the median followed by the interquartile range in parenthesis for Body Mass Index (BMI) and Metabolic syndrome (MetS). Arm circumference is shown as the median followed by the range data enclosed within parentheses OPN (ng/mg of protein).

Based on the study conducted by Cook et al., (2003), where they established criteria factors to diagnose MetS in adolescents, we re-categorized the population studied into two groups: adolescents with MetS and adolescents without MetS [29]. We observed a statistically significant increase in OPN concentration in adolescents with MetS (p = 0.04; Table 4). Arm circumference is yet another screening measure to identify obesity and hypertension, however, cutoff points have not been stablished to mestizo-Mexican adolescents or Latin-American adolescents. Arm circumference measurements from Huejutla High School adolescents were distributed and compared among tertiles and statistically significant difference was observed between the first and the third tertile Table 4.

Other parameters with significant differences in the MetS group were the BMI z-score, arm circumference, and the waist-size index. Furthermore, this group showed a low creatinine concentration, but a high TGs/Glucose ratio suggesting insulin resistance Table 5.

**Table 5:** Parameters evaluated in adolescents with metabolic syndrome from Huejutla de Reyes, Hidalgo State, Mexico.

	Without Metabolic Syndrome	Metabolic Syndrome	p-value
**Anthropometric parameters**			
BMI-z score	-0.77 (-1.45–0.202)	0.32 (-0.1009–1.03)	0.0001
Arm Circumference (cm)	25 (24–28)	29.65 (27.5–31)	0.0018
Waist-size index	0.45 (0.42–0.53)	0.58 (0.54–0.6)	0.0001
**Biochemical blood parameters**			
Total Cholesterol (mg/dl)	135.5 (127.5–155)	156 (133–170)	0.0975
Urea (mg/dl)	24 (20–32)	21 (16–22)	0.0875
Creatinine (mg/dl)	0.86 (0.81–0.95)	0.78 (0.7–0.84)	0.0088
TGs/Glucose	8.23 (7.96–8.4)	8.77 (8.2–8.89)	0.002
Chol/TGs	1.56 (1.075–1.68)	1.01 (0.92–1.28)	0.0384

### 3.3. OPN correlations

**Fig 1:**
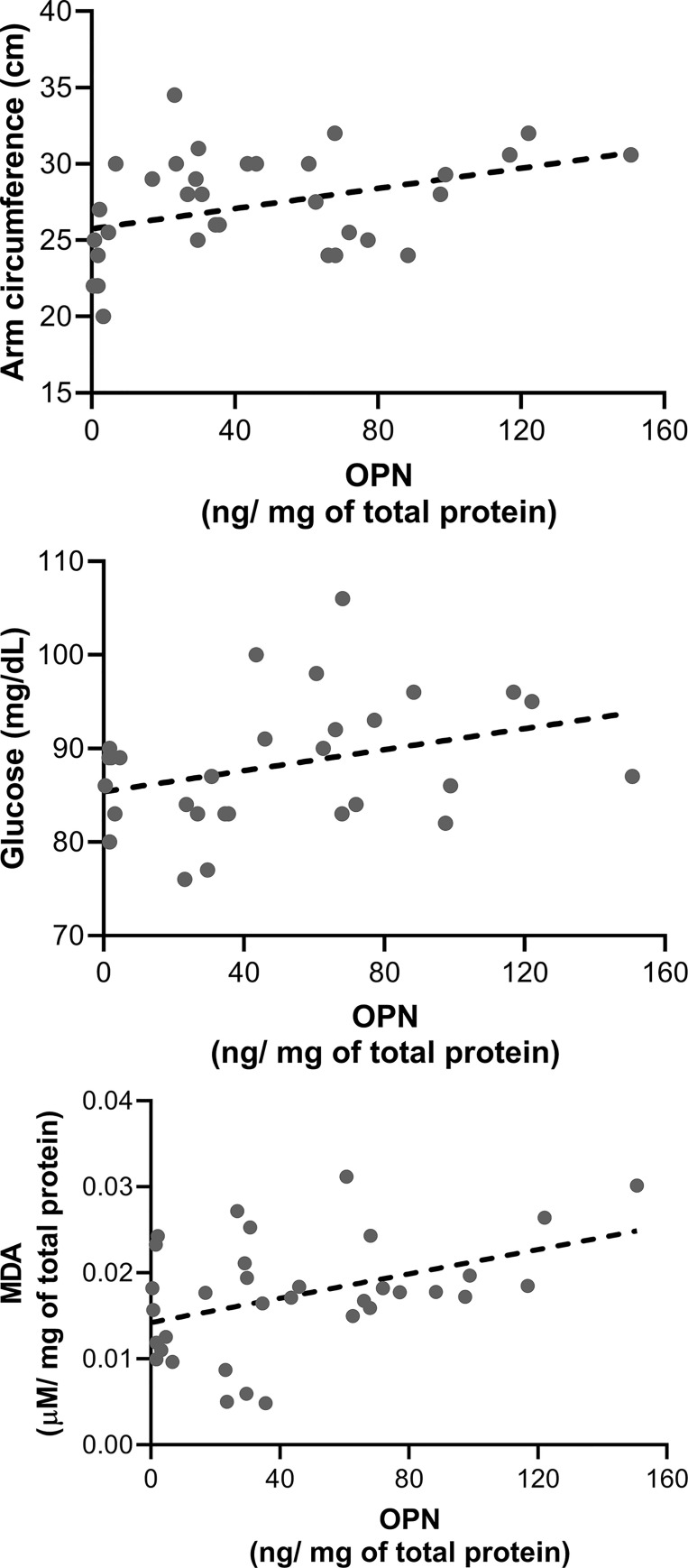
Pearson’s correlation between serum osteopontin and lipid peroxidation marker, glucose, arm circumference, in adolescent’s group. A) Relationship between serum OPN and arm circumference (ρ = 0.3412, p = 0.0449, n = 35); B) OPN and glucose showed a marginal correlation (rho 0.36, p = 0.057), and C); OPN and MDA showed a statistically significant correlation (ρ = 0.3552, p 0.0363, n = 35).

Correlation tests were performed to describe the relationships among the parameters evaluated and OPN concentrations. We observed a significant correlation of OPN with arm circumference (rho 0.407, p = 0.0169); Fig 1A but not with typical anthropometric nutritional measurements such as BMI z-score (rho 0.2139, p = 0.2244), waist circumference (rho 0.0728, p = 0.6823), weight (rho 0.0772, p = 0.6644) or size (rho -0.2407, p = 0.17). On the other hand, non-biochemical parameters analyzed showed no significant correlations with OPN concentrations, whereas, fasting blood glucose showed a marginal correlation with OPN (rho 0.36, p = 0.057, Fig 1B). With respect to the OxS biomarkers evaluated, we observed statistically significant correlation of lipid peroxidation biomarker, MDA, with OPN concentrations Fig 1C.

## 4. Discussion

Nowadays, the lack of physical activity, the increment in the consumption of a high-fat, high-calorie meal as well as highly processed food, are included in what is called obesogenic environment that contributes to weight gain in the entire population. The early beginning of weight gain in childhood predispose to development of non-communicable diseases, such as sleep apnea syndrome, asthma, nonalcoholic fatty liver disease, atherosclerosis, insulin resistance, diabetes mellitus, orthopedic problems that lead to social discrimination. As a result of high caloric intake, the growing adipose tissue due to triglycerides accumulation induce a proinflammatory state and the increment in reactive oxygen species inducing an OxS and a low chronic inflammatory condition.

OPN concentrations has been associated with tissue remodeling, fibrosis, inflammation and cancer in organs such as the lung, the heart, the kidney, the liver and the nervous system. All these pathological conditions have been studied in patients with established illness and a degree of progressive disease [30]. It has been reported an association of serum OPN levels with asthma, T1DM, kidney diseases and cancer during the childhood. However, the association of serum OPN concentration with the adipose mass condition and serum oxidative stress biomarkers has not been, to our knowledge, explored in Mexican adolescents.

It has been stablished different indexes and cutoffs to evaluate the adipose tissue and corporal mass distribution, among indexes the most frequently used are the BMI, the waist-hip ratio, the waist-size index, the abdominal and arm circumferences; all of them have differences between sex and among ages. In this study, statistical differences by sex in all parameters evaluated were not observed, however, the median serum OPN concentration showed a trend (p = 0.107) towards an increment in boys (70.05 ng/mg of total protein) compared to girls (30.33 ng/mg of total protein). Other authors indicate the importance of sex in the OPN pathological participation. Coculescu et al. found that male patients suffering from diastolic cardiac dysfunction, had higher OPN concentrations compared to females, suggesting that OPN concentrations could be determined by hormones [31]. In a rat model, Yagisawa et al. demonstrated the participation of sex hormones in the modulation of OPN kidney expression and the promotion of stone formation [32].

Adolescents were categorized by BMI, according with WHO criteria. Statistical differences were observed in the abdominal and arm circumferences, SBP, creatinine and OPN concentrations. It is well documented the relationship between the overweight and obesity with the increment in abdominal and arm circumferences and how weight gain is involved in the increment of blood pressure. Interestingly the median OPN concentrations were statistically significant in overweight and obese adolescents, as we expected. This result could suggest an inflammatory state related with such conditions and an increased cardiometabolic risk which is also related to an earlier onset and longer duration of obesity [33].

In addition, serum creatinine concentrations were decreased in obese adolescents compared with normal weight adolescents. It has been suggested that serum creatinine levels depend on age and they could be attributed to blood volume, which is relatively large in proportion to the muscle mass during this period of age [34]; however, there are not differences in age among groups classified according to BMI in this study. In this sense, we recategorize adolescents by MetS, according with the Cook et al., criteria [29], we observed that adolescents with MetS showed lower serum creatinine concentrations compared with those without MetS. These results contrast with those reported by Kelishadi et al., where they found a significant decrease in mean of serum creatinine in obese adolescents without MetS suggesting that, obesity and MetS contribute to chronic kidney diseases [35]. In addition to our study, MetS group, showed an increased BMI-z score, Waist-size index, TGs/Glucose ratio, variables that agree with the presence or insulin resistance and the low creatinine values are probably due to the increased transcapillary hydraulic pressure difference seeing in obesity [36]. Moreover, data obtained from the Kansai Healthcare Study, indicates that, lower serum creatinine was associated with an increased risk of type 2 diabetes [37]. In addition, median OPN serum concentrations were higher in MetS group, suggesting an increment of the inflammatory condition of this group that could modify the membrane permeability and an early biomineralization of soft tissues as has been indicated in different diseases [38]. Additionally, the increment observed in the BMI z-score, arm circumference, and waist-size index, are also considered as adiposity indicators and are useful tools to predict lipidic cardiometabolic risk factors [39, 40].

On the other hand, many methods to evaluate insulin resistance have been described, being the homeostasis model assessment of insulin resistance (HOMA-IR) the most used. Nevertheless, this method is based on the quantification of insulin which is expensive and not always available and/or affordable in primary health care settings. Because of this reason, other methods have been proposed as an alternative to evaluate insulin resistance such as triglyceride/glucose index. In our study we found statistically significant differences between groups, (TGs/Glu > 8.2) and an increment in the low-density particles evaluated by the total serum cholesterol/triglyceride ratio (Chol/TGs < 1.5), both factors are related with MetS and predict insulin resistance and the presence of small dense LDL particles that increase the risk of developing type 2 diabetes mellitus and cardiovascular disease respectively in children and adolescents [19, 41].

Arm circumference showed statistical difference when adolescents were grouped by BMI and the presence of MetS, we divided in tertiles the adolescent population because there are not stablished cutoff points in Mexican children. The highest median OPN concentration was observed in the last tertile of arm circumference and it was statistically significant difference compared with the first tertile, but any other biochemical parameter showed statistical difference, suggesting a relationship of OPN serum concentration with the augment in non-abdominal fat deposits. The General High School of UAEH, Huejutla de Reyes, Hidalgo State, Mexico is a School center that admits students of indigenous communities near and from Huejutla city center. Amerindian ethnic groups are comprised of Huasteca, Tének, and Náhuatl. Seventy-three percent of the inhabitants speak the native dialect Náhuatl [42]. The cultural and ethnic structure lead us to believe that adiposity in Amerindian ethnic groups influence the well documented anthropometric parameters. This observation has been reported by other studies that have described the influence of ethnicity on adiposity and risk of diabetes [43, 44].

OPN levels correlated with oxidative stress biomarker of lipid oxidation, MDA and arm circumference, but the correlation with glucose was marginal, suggesting that OPN respond to oxidative damage of lipids and to the accumulation of adipose tissue in children’s arm. Moreover, we did not see any difference in the activity of antioxidant enzymes suggesting an imbalance in the redox state increasing the OxS.

Obesity is a state of chronic OxS in which there is not clear if redox imbalance is a trigger or a result of such condition, the imbalance derived mainly from an obesogenic environment has also been associated to insulin resistance and comorbidities like diabetes, stroke, hypertension, liver disease and cancer. Studies have associated OPN levels with the expression of proinflammatory cytokines, chemokines and in the migration of monocytes/macrophages [45, 46]. Although OPN is absent in most normal soft tissues, its presence has been demonstrated at sites of atherosclerotic lesions and enhance platelet-derived growth factor (PDGF) in cultured rat aortic smooth muscle cells [4, 38], indicating ectopic calcification and favoring the development of atherosclerosis. On the contrary, the OPN deficiency has improved glucose tolerance and reduced IR in mice [47, 48].

One interesting finding was that OPN levels are higher in overweight compared to obese adolescents, probably because overweight is an initial status of inflammation and marks the beginning of metabolic disorders compared to an obesity condition where chronic pathological processes have been established. Immunological differences between overweight and obese patients have been reported in molecular expression profiles of natural killer cells, suggesting different immunological responses between overweight and obese subjects with a decline in immunological response in the obese condition [49].

The increase in OPN levels observed in mestizo-Mexican adolescents with MetS and its correlation with MDA, a reactive aldehyde involved in the pathogenesis of atherosclerosis, let us suggest that OPN could be an early indicator of vascular injury in overweight and obese adolescents that increase the risk of hypertension and atherosclerotic lesions later in adulhood. Nevertheless, further large trials are needed to validate the usefulness of serum OPN as an early risk biomarker of cardiovascular disease in obese Mexican adolescents.

Childhood obesity is associated with higher risk of disability and premature death in adulthood. Obese children have more chance to stay obese into the adulthood and to develop noncommunicable diseases, thus, serum OPN studies need to include a multifactorial approach to establish the influence of other comorbidities associated with obesity on this metabolite.

## 5. Conclusions

Our findings suggest that OPN responds to malnutrition and OxS conditions in obese mestizo-Mexican adolescents. We suggest that serum OPN could be an early biomarker to predict advanced-associated metabolic and cardiovascular complications observed in adulthood, such as diabetes mellitus and cardiovascular diseases.

## Supporting information

S1 File (TXT)
